# Analysis of factors related to short-term prognosis in patients undergoing percutaneous coronary intervention for acute myocardial infarction

**DOI:** 10.3892/etm.2013.927

**Published:** 2013-01-25

**Authors:** YI MA, LI LI, XIAO-MING SHANG, ZHENG TAN, XUE-BIN GENG, BI-QIONG ZHAO, MEI-RONG TIAN

**Affiliations:** Department of Cardiovascular Medicine, The Tangshan Affiliated Clinical Hospital of Hebei Medical University, Tangshan, Hebei 063000, P.R. China

**Keywords:** myocardial infarction, percutaneous coronary intervention, short-term prognosis, risk factors

## Abstract

The present study aimed to investigate the factors related to short-term prognosis in patients undergoing direct percutaneous coronary intervention (PCI) for acute ST-segment elevation myocardial infarction (STEMI). A total of 805 patients were included and divided into a control group and an adverse cardiovascular events group based on the prognosis, to compare risk factors and coronary angiographic characteristics in the two groups. In the adverse events group, the ages, admission blood glucose, uric acid (UA), homocysteine (HCY), creatine kinase (CK) and peak creatine kinase-MB (CKMB) isozyme levels were clearly higher compared with those in the control group, while the levels of total cholesterol (TC), triglycerides (TGs) and low-density lipoprotein (LDL) were lower compared with those in the control group. The incidence of hypertension in females in the adverse events group was markedly higher compared with that in the control group, while the diabetes rate was lower compared with that in the control group. Logistic regression analysis revealed that age, gender, hypertension, diabetes and admission blood glucose, HCY, TC and UA levels were independent risk factors of short-term prognosis in patients undergoing emergency PCI. The majority of the patients in the adverse events group were elderly females with hypertension, a large area of myocardial infarction and increased admission blood glucose, UA and HCY levels, as well as a low diabetes rate and decreased levels of acute-phase TC and LDL.

## Introduction

Acute myocardial infarction (AMI) is a common disease that is harmful to human health and causes mortality in patients. In the United States, which has a high incidence, ∼1.5 million individuals suffer from AMI each year. With the development of the economy and lifestyle changes, the incidence of AMI in China has increased and AMI has become the main cause of mortality. AMI is the most critical cardiovascular disease with high lethality and disability rate. Percutaneous coronary intervention (PCI) is currently the preferred treatment for ST-segment elevation myocardial infarction (STEMI), however, various factors affect its prognosis. A study has shown that with increasing age, the morbidities of hypertension and old myocardial infarction in AMI patients are significantly increased and the incidence rates of mechanical complications, arrhythmia and multivessel disease are also clearly higher than in younger patients (1,2). The hospital mortality rate of female AMI patients is significantly higher than that of males (3,4) and the increase of serum uric acid (UA) (5–7) and homocysteine (HCY) (8) increases the morbidity and mortality of cardiovascular diseases. Age, gender, blood pressure, lipid level and blood glucose are predictors influencing the outcome of AMI. Genetic factors, living conditions and eating habits are also risk factors with regional differences. In this study, 805 patients with AMI successfully received direct PCI therapy. The risk factors, clinical features and lesion characteristics of the coronary artery were compared between the adverse events group and the control group. The risk factors of adverse events with an onset of 30 days were subjected to logistic regression analysis to provide clues for identifying high-risk patients in the early stage and reduce the mortality of AMI patients through early intervention.

## Patients and methods

### Patients

This study was conducted in accordance with the Declaration of Helsinki and with approval from the Ethics Committee of the Tangshan Affiliated Clinical Hospital of Hebei Medical University. Written informed consent was obtained from all participants. From January 2009 to February 2011, 813 patients with STEMI that attended our hospital were continuously collected. They all received direct PCI within 12 h of the onset of symptoms. Eight cases were lost to follow-up; therefore, 805 patients were included in the study. AMI is diagnosed if the patient fulfills two of the following three standards: i) chest pain continuing for >30 min; ii) dynamic evolution of typical ST-T appears in the electrocardiogram and iii) dynamic increases in troponin I, troponin T and CK isozyme levels to >2-fold higher than the normal upper limit. The exclusion standards are: i) cardiogenic shock; ii) ventricular septal perforation and fractured chordae tendineae of the mitral valve or papillary muscles; iii) infarct-related artery (IRA) has a left main coronary artery disease or other coronary occlusion with severe stenosis of the left main coronary artery; iv) IRA is in the presence of >2 collateral circulations and v) AMI is caused by an interruption to the coronary blood flow as a result of invasive diagnosis and treatment or other diseases. All patients received coronary angiography and conventional multi-position projection within 12 h of the onset to determine the number of diseased coronary arteries and IRA for PCI. Patients were treated with 0.3 g aspirin and 300 mg Plavix preoperatively and a subcutaneous injection of low molecular weight heparin postoperatively, as well as a IIb/IIIa receptor antagonist, aspirin, Plavix, angiotensin-converting enzyme inhibitors, β-receptor blockers and nitrate esters, according to the guidelines. PCI was considered successful if the distal blood flow was grade 3 according to the Thrombolysis in Myocardial Infarction (TIMI) Study Group grading system with <20% residual stenosis in the IRA.

### Grouping

The patients were divided into an adverse events group and a control group according to whether they presented angina pectoris (with ischemic electrocardiographic ST-T changes), re-infarction (coronary angiography confirmed in-stent acute and subacute thrombotic occlusion), heart failure (Killip class >II), cardiac rupture or mortality within 30 days of AMI.

### Observation indices

The general information and AMI risk factors in the groups were compared and analyzed, including age, hypertension, hyperlipidemia, diabetes, total cholesterol (TC), triglycerides (TGs), high-density lipoprotein, low-density lipoprotein (LDL), blood UA, HCY and admission blood sugar. Other prognostic factors included time from symptom onset to PCI and the number of IRA and diseased lesion vessels.

### Statistical analysis

The enumeration data in the two groups were compared by 2×2 Chi-square test. The measurement data were determined by two-sample t-test. Logistic regression analysis was applied to screen the independent risk factors influencing the short-term prognosis in patients undergoing PCI for AMI. Statistical analysis was carried out using the SPSS 16.0 for Windows software package (SPSS Inc., Chicago, IL, USA). P<0.05 was considered to indicate a statistically significant difference.

## Results

### General data

Among the 805 patients, there were 144 patients in the adverse events group, including 45 cases of postoperative angina pectoris with ischemic electrocardiographic ST-T changes, 32 cases of re-infarction confirmed by coronary angiography as in-stent acute and subacute thrombosis, 59 cases of heart failure, 12 cases of cardiac rupture and 44 cases of mortality. There were 661 patients in the control group. Compared with the control group, patients in the adverse events group were older and the prevalence of female hypertension was significantly higher (P<0.05); however, there was a low rate of diabetes, with no statistical significance. There were no significant differences in time from symptom onset to PCI, hospital heart rate, systolic blood pressure and diastolic pressure in the two groups; however, the instant glucose level, UA and serum HCY levels at admission were clearly higher than those in the control group. Additionally, the peak levels of creatine kinase-MB (CKMB) enzyme detected during hospitalization were significantly higher in the adverse events group (P<0.05). The levels of TC and LDL detected in the morning of the day following admission to the hospital were significantly lower in the adverse events group than those in the control group (P<0.05). Coronary angiography results demonstrated that in the adverse events group, the proportion of patients with an infarction-associated left circumflex coronary artery (LCX) was significantly lower than that in the control group and the proportion of patients with an infarction-associated left anterior descending artery (LAD) was higher than that in the control group,.without a statistical significance. The proportions of patients with right coronary artery (RA) infarction were almost equal in the two groups. In the adverse events group, the ratio of patients with single-vessel diseases was almost equal to that of the control group. In the adverse events group, the incidence of patients with 2-vessel diseases was lower than that in the control group; however, the incidence of patients with 3-vessel diseases was significantly higher than that in the control group (P<0.05; Table I).

### Logistic regression analysis

The combined results of mortality, re-infarction, angina pectoris, heart failure, cardiac rupture and other adverse events within 30 days after AMI were considered as the dependent variables (negative=1; positive=2) and age, hypertension, diabetes, TG, LDL, blood UA, HCY, admission blood glucose, infarct-related blood vessels and the number of diseased blood vessels were considered as the independent variables. We observed that age, gender, hypertension, diabetes, admission blood glucose, HCY, TC and UA level were the independent risk factors of short-term prognosis in patients undergoing PCI for AMI (Table II).

## Discussion

In this study, we analyzed the clinical characteristics, risk factors and coronary artery lesion characteristics of patients undergoing PCI for AMI over a two-year period. Previous studies have reported that the correlation between hyperglycemia and the prognosis of patients with AMI may be studied by selecting the instant hospital blood glucose value or blood glucose within 24 h (9–11). Acute hyperglycemia is a risk factor for poor prognosis of AMI (12) and an independent risk factor of increased mortality of AMI patients (13). The elevated blood glucose level not only reflects the acute stress state, but also increases the risk of cardiovascular events. Consistent with previous studies, the data in the current study highlights the importance of blood glucose in the occurrence of adverse events. The admission glucose levels of patients in the adverse events group were significantly higher than those in the control group. We assessed the independent risk factors of adverse events by logistic regression analysis and identified that the risk of adverse events in patients with an instant blood glucose >11.1 mmol/l was 6-fold higher than that in patients with an instant blood glucose <11.1 mmol/l. However, the proportion of cases of diabetes in the adverse events group was clearly lower than that in the control group, but without a significant difference. Diabetes was negatively correlated with adverse events and the proportion of nondiabetics was 1.8-fold higher than that of diabetics. The greater risk of adverse events in non-diabetic patients than in diabetic patients may be due to non-diabetic AMI patients presenting acute hyperglycemia in addition to stress. In a prospective study (14), AMI patients without a history of diabetes and admission blood glucose <11.1 mmol/l underwent an oral glucose tolerance test and 40% had abnormal glucose tolerance and 25% had previously undiagnosed diabetes. A study of the abnormal glucose metabolism in acute coronary syndrome patients demonstrated that the ratio of blood glucose dysregulation in non-diabetics and newly diagnosed diabetics reached 40 and 22%, respectively (15). Compared with diabetic patients that have been diagnosed, the non-diabetics did not have well-controlled hyperglycemia, which increased endothelial damage and caused microvascular and macrovascular disease. Therefore, non-diabetics have an increased risk of adverse events.

In the current study, the blood lipid levels of the patients with AMI were detected in the morning of the day after AMI and we observed that the levels of TC and LDL in the adverse events group were significantly lower than those in the control group. Cholesterol was identified as an independent risk factor for adverse events by logistic regression analysis and the TC level was negatively correlated with adverse events. There are few reports concerning the effect of blood lipid levels on the short-term prognosis of AMI. Gore *et al* (16) reported that in the early stages of AMI, the TC levels of patients were significantly reduced. Fyfe compared blood TC and TG levels in 50 cases of AMI at admission (within 24 h of onset) and 3 months after discharge (17). The authors identified that TC was significantly reduced at admission and the decline range and time-frame were related to the degree of heart disease. In non-fatal myocardial infarction, TC declined by 18% and in lethal myocardial infarction it declined by 26%. It was considered that in the acute period of AMI, the decrease of blood lipid level may be related to an acute stress response and inflammatory reaction, inhibiting the expression and secretion of apolipoprotein B (ApoB) mRNA in liver cells, thus inhibiting the synthesis of TC and TG (16,18).

Consistent with previous reports (8,19–24), our data demonstrated that in the adverse events group the ages of the patients were higher than those in the control group and the proportion of females was significantly higher than that in the control group. The morbidity of hypertension in the adverse events group was higher than that in the control group and UA, HCY and CK levels, as well as CKMB enzyme peak were clearly higher than those in the control group. Using coronary angiography, we identifed that the incidence rate of single-vessel coronary artery diseases was similar in the two groups; however, the incidence rate of 2-vessel diseases was significantly lower and the incidence rate of 3-vessel diseases was signficantly higher in the adverse events group. Infarct-related blood vessels in the RA were similar in the two groups and the incidence rate of LCX infarction was significantly lower in the adverse events group. The incidence rate of the LAD infarction was higher than that in the control group, although the difference was not statistically significant. Using logistic regression, we identified that age, female gender, hypertension, UA and HCY levels are independent risk factors of adverse events and are positively correlated with the incidence of adverse events. The risk of adverse events in patients aged >60 years was 1.9-fold higher than the risk in those aged <60 years. The risk of adverse events in female patients was 2.1-fold higher than the risk in male patients and the risk of adverse events in hypertensive patients was 1.9-fold higher than the risk in non-hypertensive patients. The risk of adverse events in patients with hyperuricemia was double that in patients with normal UA and the risk of adverse events in patients with hyperhomocysteinemia was 2.6-fold higher than the risk in normal patients.

According to the results in this study, there were more elderly females with hypertension in the adverse events group; however, there was also a low rate of diabetes, more 3-vessel coronary artery diseases, a greater proportion of patients with a large myocardial infarction area and significantly higher admission blood glucose, UA and HCY levels than in the control group, as well as significantly decreased TC and LDL levels in the acute phase.

This study was a single-center trial with a small sample size and, due to the high treatment cost of direct PCI following AMI, the results were limited by the patients’ economic conditions. In future studies, the short follow-up period should be extended to predict the patient’s risk more thoroughly.

## Figures and Tables

**Table I:** Comparison of the clinical characteristics between groups.

Characteristics	Adverse events group (n=144)	Control group (n=661)	t/χ^2^ value	P-value
Age (years)	62.97±10.912	58.83±10.609	4.223	0.00
Females [n (%)]	40 (27.8)	92 (13.9)	15.572	0.00
Hypertension [n (%)]	80 (55.6)	287 (43.5)	7.021	0.008
Diabetes [n (%)]	28 (19.4)	164 (24.8)	1.875	0.171
Hyperlipidemia [n (%)]	83 (57.6)	401 (60.7)	0.452	0.501
Disease onset to PCI				
Time (h)	5.01±2.948	5.04±2.711	0.113	0.910
Heart rate (bpm)	81.56±19.92	81.33±16.59	0.140	0.889
SBP (mmHg)	121±18	124±16	1.861	0.063
DBP (mmHg)	75±10	75±10	0.504	0.614
Infarction-associated blood vessels				
LAD [n (%)]	71 (49.3)	284 (43.0)	1.928	0.165
LCX [n (%)]	12 (8.3)	105 (15.9)	4.838	0.028
RA [n (%)]	61 (42.4)	272 (41.2)	0.072	0.789
Single-vessel lesion [n (%)]	52 (36.1)	245 (37.1)	0.046	0.830
Double-vessel lesion [n (%)]	44 (30.6)	280 (42.4)	6.851	0.009
Triple-vessel lesion [n (%)]	48 (33.3)	132 (20.0)	11.406	0.001
TC (mmol/l)	4.598±0.820	4.848±1.020	2.748	0.006
LDL (mmol/l)	2.666±0.817	2.928±0.882	3.274	0.001
Admission blood glucose (mmol/l)	7.457±2.948	6.785±2.620	2.723	0.007
CK enzyme peak (U/l)	2314.3±1238.6	1940.4±1550.9	2.710	0.007
CKMB enzyme peak (U/l)	213.22±122.19	166.42±110.23	4.525	0.000
UA (*μ*mol/l)	334.25±94.56	311.18±93.59	2.675	0.008
HCY (*μ*mol/l)	23.59±12.410	18.51±11.117	4.885	0.000

PCI, percutaneous coronary intervention; SBP, systolic blood pressure; DBP, diastolic blood pressure; LAD, left anterior descending artery; LCX, left circumflex coronary artery; RA, right coronary artery; TC, total cholesterol; LDL, low-density lipoprotein; CK, creatine kinase; CKMB, creatine kinase-MB; UA, uric acid; HCY, homocysteine.

**Table II:** Logistic regression analysis outcomes.

Factors	β-value	SE-value	OR (95% CI)	P-value
Gender	0.723	0.259	2.061 (1.240–3.424)	0.005
Age	0.632	0.229	1.881 (1.201–2.947)	0.006
Hypertension	0.650	0.210	1.915 (1.269–2.892)	0.002
Diabetes	−0.608	0.264	0.545 (0.325–0.913)	0.021
Cholesterol	−0.731	0.247	0.481 (0.296–0.781)	0.003
Uric acid	0.707	0.286	2.028 (1.159–3.551)	0.013
Homocysteine	0.942	0.228	2.566 (1.640–4.014)	0.000
Blood glucose	1.787	0.216	5.974 (3.913–9.120)	0.000

SE, standard error; OR, odds ratio; CI, confidence interval.
